# Language Models as a Challenge for Business Ethics – A Partially Open-Source Approach

**DOI:** 10.1007/s11948-026-00620-0

**Published:** 2026-08-14

**Authors:** Patrick Hedfeld

**Affiliations:** FOM and Goethe University, Theodor W. Adorno Platz, Frankfurt am Main, 60323 Hessen Germany

**Keywords:** Large language models, AI governance, Business ethics, Institutional economic ethics, Karl Homann, Transparency, Linguistic inequality, Data governance, Partially open AI

## Abstract

Large language models have become central infrastructures of contemporary digital economies while raising persistent ethical concerns regarding linguistic inequality, opacity, data governance, and the concentration of technological power. Much of the current debate on AI ethics focuses on normative principles such as fairness, transparency, and accountability. While these principles remain essential, they often do not sufficiently explain why ethically problematic outcomes persist under competitive market conditions. This paper addresses that gap by applying Karl Homann’s institutional economic ethics to the governance of large language models. From this perspective, ethical deficits in AI development are not merely the result of individual failures of responsibility, but are also shaped by institutional incentive structures that reward speed, scale, proprietary control, and strategic secrecy. The paper analyses three central ethical challenges in LLM development: linguistic and cultural asymmetries, transparency and accountability deficits, and contested practices of data governance and intellectual property. It then argues that the familiar opposition between open and closed AI systems is conceptually and institutionally inadequate. In response, the paper develops the concept of partially open AI governance, understood as a differentiated arrangement of access, disclosure, and oversight across distinct layers of AI systems. Such an approach offers a more realistic way of aligning innovation incentives with ethical and public objectives in the governance of large language models.

## Introduction

### Problem Outline: AI, Language, and Cultural Embedding

Large language models (LLMs) have become a central technological infrastructure in contemporary digital societies. Their rapid development has transformed the ways in which language is processed, generated, and distributed across communication platforms, search engines, educational settings, and professional environments. Since systems such as GPT-3 and related models have demonstrated that scaling model size, data, and computational resources can produce substantial gains in performance, LLMs have increasingly been perceived not merely as experimental tools but as economically strategic technologies with broad societal relevance (Brown et al., 2020).

This development has intensified ethical debates about the role of artificial intelligence in shaping public communication, knowledge production, and access to information. Because language models are trained on very large corpora of human-generated text, they do not operate in a socially neutral environment. Rather, they reproduce patterns, hierarchies, and asymmetries already embedded in linguistic and cultural data. Concerns about biased outputs, uneven language representation, and the amplification of dominant discursive patterns therefore occupy a prominent place in the emerging literature on LLM ethics (Daly et al., 2021; Ferdaus et al., 2024). These concerns are particularly relevant in the case of globally deployed models, which often reflect the predominance of English-language and high-resource data environments while underrepresenting less commercially valuable linguistic contexts.

At the same time, ethical concerns about large language models extend beyond bias and cultural representation. Questions of transparency and accountability have become equally pressing. Many of the most influential models are developed within highly concentrated corporate environments, where the technical architecture, training data, and evaluation procedures remain only partially disclosed. This creates significant obstacles for external scrutiny and for public assessment of how these models function, what data they rely on, and what kinds of harms they may reproduce. The opacity of such systems becomes especially problematic when language models are integrated into domains with social, economic, or political relevance.

The growing importance of large language models is therefore accompanied by a broader governance problem. Their development is not only a technical matter but also a question of how linguistic infrastructures are shaped by economic interests, institutional rules, and competitive strategies. Firms developing LLMs operate under strong pressures to innovate rapidly, protect proprietary advantages, and secure market positions in an increasingly competitive environment. As a result, many of the ethical problems associated with LLMs cannot be understood solely as failures of individual responsibility. They are also outcomes of the institutional conditions under which these systems are developed and deployed.

### Research Question: Economic Incentives, Fairness, and Transparency

Much of the current debate on AI ethics is organized around normative principles such as fairness, transparency, accountability, and responsibility. These principles are indispensable for evaluating the societal implications of artificial intelligence. However, they are often discussed at a level of abstraction that does not sufficiently account for the economic and institutional dynamics shaping the production of AI systems. In the case of large language models, this limitation is particularly important. The development of advanced models requires substantial investments in computational infrastructure, training data, technical expertise, and organizational capacity. These conditions favor firms with extensive financial and technological resources and create incentives to protect strategic assets through closed development models.

From this perspective, key ethical tensions in LLM development can be understood as conflicts between commercial rationality and broader societal expectations. Firms have strong incentives to limit disclosure of model weights, training data, and technical procedures in order to maintain intellectual property, avoid imitation, and secure competitive advantages. Yet this same opacity undermines transparency, reduces accountability, and makes it more difficult to assess bias, exclusion, or harmful downstream effects. Conversely, more open approaches may improve scrutiny and collaborative innovation, but they can also weaken business incentives and raise concerns about misuse, safety, and the uncontrolled diffusion of powerful systems (Langenkamp & Yue, 2022; Khanjani & Sulaiman, 2011).

The central problem is therefore not simply whether firms behave ethically or unethically. Rather, the decisive question is how the institutional and competitive environment of AI development shapes the incentives under which firms make strategic decisions. If ethical concerns such as fairness, linguistic inclusion, and transparency generate costs without producing corresponding competitive advantages, firms cannot be expected to prioritize them consistently under market conditions. In such cases, ethical deficits are better interpreted as structurally induced outcomes rather than as mere moral failures (Jourdan, 2024).

Against this background, this paper asks: **How do the institutional incentive structures of large language model development shape ethical challenges such as fairness**,** transparency**,** and data governance**,** and what form of AI governance could better align innovation incentives with socially desirable outcomes?** This question shifts the focus from purely normative evaluation to an analysis of the rules, incentives, and governance arrangements that condition AI development. It also allows the paper to address the growing tension between closed proprietary systems and calls for greater openness in AI ecosystems.

The argument pursued in this paper is that the debate over open versus closed AI systems should not be treated as a binary opposition. Instead, the ethically relevant issue concerns how different degrees of openness affect innovation incentives, accountability, and the distribution of risks and benefits. On this basis, the paper develops the idea of a partially open governance model for large language models, designed to reconcile the economic interests of developers with the ethical need for greater transparency and public oversight.

### Theoretical Approach: Karl Homann’s Institutional Ethics

To address this problem, the paper draws on Karl Homann’s institutional economic ethics. Homann’s approach begins from the premise that modern market societies are structured by competition and that individual actors, including firms, typically cannot be expected to realize ethical objectives at their own expense on a stable basis (Homann & Blome-Drees, 1992; Homann & Suchanek, 2005; Homann, 2004; Boutilier et al., 1997). Under competitive conditions, actors are pressured to pursue strategies that secure economic survival and success. Ethical expectations directed at individual firms therefore remain fragile if they are not supported by institutional rules that alter incentive structures.

This perspective is particularly relevant for the governance of large language models. The ethical concerns associated with LLMs—such as opacity, linguistic inequality, exclusion, or problematic data practices—arise in an environment in which firms face strong incentives to scale rapidly, secure proprietary advantages, and minimize disclosure. In such a setting, voluntary commitments to fairness or transparency are often unstable, especially when they conflict with competitive interests. Homann’s framework suggests that the relevant level of analysis is therefore not primarily the moral disposition of individual organizations but the institutional configuration within which they operate.

An institutional economic perspective also helps to clarify why governance debates about artificial intelligence should focus on the alignment of incentives rather than on abstract ethical demands alone. If markets reward speed, scale, and exclusivity while imposing few effective obligations regarding transparency or linguistic inclusion, ethically problematic outcomes are likely to persist even when firms publicly endorse ethical principles. For Homann, the task of ethics in market contexts is therefore to design rules of the game in such a way that socially desirable conduct becomes compatible with rational self-interest. This does not eliminate moral responsibility, but it shifts analytical attention toward the institutional conditions that make responsible conduct more or less likely.

Applying this framework to large language models allows the paper to reinterpret current governance conflicts in a more systematic way. The tension between proprietary control and public accountability, for instance, can be analyzed as an institutional dilemma rather than merely as a disagreement about corporate virtue. Similarly, debates over open-source AI can be examined in terms of how different governance arrangements distribute incentives, costs, and risks across firms, regulators, and affected publics. This opens a path toward a more realistic ethical analysis of AI development—one that takes seriously both the normative concerns surrounding LLMs and the competitive dynamics that shape the behavior of actors in this field.

The paper’s contribution is threefold. First, it applies Homann’s institutional economic ethics to the governance of large language models and thereby connects debates in AI ethics with questions of competitive order and incentive design. Second, it examines key ethical challenges in LLM development, with particular attention to linguistic asymmetries, transparency deficits, and data governance. Third, it develops the concept of partially open AI governance as a possible institutional response to the tension between innovation incentives and ethical demands for accountability. The remainder of the paper proceeds by first outlining the institutional economic framework, then analyzing the economic structure of the LLM industry, and finally discussing the governance implications of different forms of openness in AI development.

## Institutional Economic Ethics and AI Governance

### Karl Homann’s Institutional Economic Ethics

Karl Homann’s institutional economic ethics offers a particularly useful framework for analysing the ethical challenges associated with large language models because it shifts attention away from the moral intentions of individual actors and towards the institutional conditions under which economic decisions are made (Homann & Blome-Drees, 1992; Homann & Suchanek, 2005). At the centre of Homann’s approach lies the insight that modern market societies are structured by competition. Firms operate under pressure to remain profitable, protect their position in the market, and respond strategically to the actions of competitors. Under such conditions, ethical expectations addressed solely to individual companies remain fragile if acting ethically entails costs that rival firms can avoid.

From this perspective, business ethics cannot be reduced to appeals to corporate virtue or goodwill. Rather, it must address the rules of the game within which firms act. Homann argues that ethical objectives in market economies can only be stabilised if they are embedded in institutions that align individual self-interest with collectively desirable outcomes (Homann, 1997; Homann & Suchanek, 2005). If socially desirable conduct, such as increased transparency or fairer access to technological benefits, systematically places actors at a competitive disadvantage, then such conduct will remain difficult to sustain under market conditions.

This approach is highly relevant in technologically dynamic sectors in which competitive pressure, innovation incentives, and strategic secrecy play a particularly strong role. In such environments, firms may well recognise the ethical significance of certain concerns, yet still refrain from addressing them adequately because doing so would generate costs or reduce their competitive advantage. Institutional economic ethics therefore does not deny the relevance of moral norms such as fairness, responsibility, or transparency. Instead, it asks under what institutional conditions these norms can become effective in practice (Pies, 2011).

For the governance of artificial intelligence, and especially large language models, this insight is crucial. The question is not merely whether firms should act responsibly, but whether the institutional environment in which they operate makes responsible conduct compatible with economic rationality. Homann’s theory is therefore particularly well suited to an analysis of AI development, where powerful ethical expectations coexist with intense market rivalry and strong incentives for proprietary control.

### Ethical Challenges of LLMs as Institutional Problems

When applied to the development of large language models, Homann’s framework makes it possible to reinterpret central ethical concerns as structurally embedded rather than merely individually caused. The development of LLMs takes place in a context characterised by high computational costs, the strategic value of data, and intense competition for market leadership. Firms developing such systems therefore face strong incentives to scale rapidly, protect their models, and prioritise commercially valuable forms of deployment. Under these conditions, ethical deficits may emerge not because firms explicitly reject ethical principles, but because the institutional setting rewards other priorities more strongly.

This can be seen, first, in the area of transparency. External scrutiny of large language models often requires access to information about architecture, training procedures, data composition, evaluation practices, and, in some cases, model weights. Yet these are precisely the forms of information that companies frequently regard as commercially sensitive. More extensive transparency can improve accountability and facilitate independent criticism, but it may also weaken proprietary advantages and expose firms to imitation or regulatory scrutiny. In competitive environments, selective opacity may therefore be a rational business strategy even when it is ethically problematic (Langenkamp & Yue, 2022; Touvron et al., 2023).

Second, similar tensions arise in relation to fairness and linguistic inclusion. A large body of debate in AI ethics has shown that language technologies can reproduce existing asymmetries in representation, visibility, and access. In the case of large language models, these asymmetries are often intensified by the economic logic of training and deployment. High-resource languages and user groups with significant commercial relevance are more likely to be represented adequately because they promise greater returns on investment. By contrast, linguistic minorities or less profitable contexts may remain underrepresented or receive less technical attention. Ethical issues such as bias and linguistic exclusion must therefore be understood in connection with the market conditions that shape the allocation of resources in model development (Daly et al., 2021; Ferdaus et al., 2024).

Third, data governance and intellectual property raise analogous institutional tensions. The performance of large language models depends heavily on access to vast amounts of textual material. This creates incentives to use extensive datasets even when the legal and ethical status of particular sources remains unclear. Questions concerning copyrighted content, authorship, consent, and provenance become especially difficult in a competitive environment in which scale is rewarded and enforcement remains limited. Here again, firms may act in ways that are economically rational under prevailing conditions while generating significant ethical and legal concerns. The underlying problem is not merely one of individual misconduct, but of incentive structures that reward rapid accumulation and deployment of data-intensive systems.

From an institutional economic perspective, these tensions reveal a general pattern. Ethical concerns become persistent when the market order fails to reward or protect socially desirable conduct. If firms incur costs for increasing transparency, for investing in multilingual robustness, or for introducing more stringent standards of data governance, while competitors can avoid those costs, then moral exhortation alone is unlikely to produce stable ethical outcomes. Homann’s theory therefore suggests that the central task is not simply to formulate better ethical principles, but to develop institutional arrangements under which compliance with such principles becomes strategically viable (Homann & Blome-Drees, 1992; Homann & Suchanek, 2005; Homann, 2004).

### Institutional Ethics and the Design of AI Governance

This institutional perspective has important consequences for the governance of large language models. It implies that AI ethics cannot be reduced to a catalogue of norms directed at firms, developers, or users. Ethical governance must instead address the broader regulatory and competitive environment in which these actors operate. The relevant question becomes how institutions can be designed so that innovation incentives are not systematically opposed to transparency, accountability, fairness, and responsible data practices.

This argument is particularly important in current debates over the openness of AI systems. Closed models offer firms stronger control over intellectual property, business strategy, and deployment risks. At the same time, they reduce external oversight and make public accountability more difficult. Open models, by contrast, may promote transparency, collaborative innovation, and broader access, but they also raise concerns relating to misuse, safety, and weakened incentives for private investment (Khanjani & Sulaiman, 2011; Langenkamp & Yue, 2022). From an institutional economic perspective, neither side of this opposition can simply be declared ethically superior in the abstract. What matters is how different governance arrangements shape incentives and distribute benefits, burdens, and risks.

Homann’s framework is useful here because it directs attention to incentive compatibility. If governance models are to be effective, they must be designed in a way that acknowledges the strategic interests of firms rather than assuming that such interests can simply be overridden by moral demands. This does not mean that regulation should defer to commercial priorities. Rather, it means that durable ethical governance depends on institutions capable of reshaping the payoff structure of market behaviour. In the field of large language models, this may involve transparency obligations, differentiated access regimes, auditing requirements, or governance structures that combine openness in some components of AI systems with restrictions in others.

The concept of partially open AI governance, which will be developed later in this paper, is intended as a contribution to precisely this problem. Instead of treating openness as a binary choice, it asks how different components of AI systems—such as model architecture, documentation, training data, or weights—might be governed under differentiated conditions. Such an approach is consistent with institutional economic ethics because it aims to reconcile competing goals rather than treating ethical demands and economic rationality as entirely separate spheres.

The next section therefore turns to the economic structure of the LLM industry itself. If ethical conflicts in AI development are shaped by institutional incentives, then it is necessary to examine more closely the market conditions under which large language models are developed, financed, and deployed. Only then can the ethical and governance problems of these systems be analysed with sufficient precision.

## The Economic Structure of the LLM Industry

### Large Language Models as Resource-Intensive Technologies

The development of large language models is shaped by a highly resource-intensive production environment. Unlike smaller-scale software systems, advanced LLMs require extensive computational infrastructure, access to large quantities of textual data, and highly specialised engineering expertise. This has important economic implications. It means that the development of powerful models is not open to all market participants on equal terms, but instead depends on access to capital, hardware, data, and organisational capacity at a scale that only a limited number of actors can mobilise (Brown et al., 2020; OpenAI, 2023).

These structural conditions influence both market entry and competitive behaviour. Training large models involves high fixed costs, while many of the expected benefits arise only if firms are able to deploy their systems at scale across a wide range of products and services. This creates strong incentives for firms to invest heavily in model performance, platform integration, and speed of commercialisation. In economic terms, LLM development is therefore characterised by substantial barriers to entry and by a tendency towards concentration among firms that can absorb large up-front costs and monetise the resulting systems through established digital ecosystems (Korinek, 2019; Michalak & Wooldridge, 2017).

The resource intensity of LLMs also affects the distribution of power within the AI sector. Companies with access to cloud infrastructure, proprietary datasets, and financially backed research teams are in a stronger position to build and refine advanced models than smaller firms, public institutions, or actors from less capitalised regions. This means that the architecture of the market itself may reinforce asymmetries in who can shape the future direction of language technologies. From the perspective of institutional ethics, this matters because ethical concerns about accountability and inclusion are connected not only to model outputs but also to the economic structure that determines who gets to build, control, and deploy such systems.

### Market Concentration, Strategic Advantage, and Proprietary Control

A key feature of the LLM industry is the concentration of strategic advantage in the hands of a relatively small number of firms. The high costs of training and maintaining advanced models create incentives for vertical integration and for the protection of proprietary assets. These assets include model weights, training pipelines, engineering practices, evaluation procedures, and access to data. Firms that control such assets can use them not only to improve model performance, but also to consolidate their market position by embedding language models into broader product ecosystems and service infrastructures (OpenAI, 2023; Touvron et al., 2023).

Under these conditions, opacity is not merely a technical or organisational choice; it is often also a market strategy. Restricting access to technical details or model components may serve to preserve competitive advantage, reduce imitation, and protect future revenue streams. At the same time, such strategies make external assessment more difficult and weaken possibilities for independent auditing or public accountability. This demonstrates that the ethical problem of opacity cannot be understood independently of the economic logic of proprietary competition.

The economic relevance of openness and closure has also been discussed in the broader literature on open-source software and AI development. Open models may lower barriers to innovation, enable collaborative improvement, and support wider participation in technical development. However, firms may still prefer more closed arrangements when they seek stronger control over appropriation, liability exposure, or strategic differentiation (Khanjani & Sulaiman, 2011; Langenkamp & Yue, 2022). In this sense, the tension between open and closed AI systems reflects a deeper conflict between different models of innovation and control. The question is therefore not only whether openness is ethically desirable, but how different degrees of openness affect competitive incentives, market structure, and the distribution of technological capabilities.

### Incentives, Externalities, and Ethical Underinvestment

The economic structure of the LLM industry also helps explain why ethical concerns often remain insufficiently addressed even when they are widely acknowledged. Many ethically desirable measures in AI development generate costs that are borne by individual firms, while the benefits are often diffuse, collective, or long term. This applies, for example, to extensive documentation, bias evaluation across diverse populations, investments in underrepresented languages, and stricter standards for dataset provenance and consent. Such measures may improve the social quality of AI systems, but they do not necessarily produce immediate competitive advantages.

This creates a pattern of ethical underinvestment. Firms operating under strong market pressure may rationally prioritise capabilities that are easier to commercialise over investments whose returns are uncertain or partly external to the firm. From an economic perspective, the result resembles a collective action problem: each actor may have incentives to minimise ethical costs in order to remain competitive, even though the aggregate outcome may be socially undesirable because harmful biases, low transparency, and weak accountability become normalised (Homann & Suchanek, 2005; Korinek, 2019).

The same logic applies to multilinguality and linguistic inclusion. Supporting a wide range of languages and sociocultural contexts requires data curation, evaluation, and model refinement that may not be commercially attractive in the short term. If market returns are concentrated in a smaller number of dominant languages, firms have incentives to allocate resources accordingly. As a result, linguistic inequality is not only a technical limitation but also an economically structured outcome. The ethical problem of exclusion is therefore closely related to the incentive system of model development rather than being reducible to accidental oversight (Daly et al., 2021; Ferdaus et al., 2024).

Questions of intellectual property and data governance fit the same pattern. The use of vast textual corpora can improve performance, but the legal and ethical status of those corpora may remain uncertain. In highly competitive markets, firms may face incentives to maximise data acquisition and model quality first and address legal or ethical concerns only later, especially when the relevant standards remain contested or weakly enforced. This does not imply that all firms act irresponsibly in the same way. It does suggest, however, that the market environment may systematically reward strategies that externalise ethical and legal risks.

### Why Industry Structure Matters for AI Governance

These features of the LLM industry have direct implications for governance. If ethical challenges such as opacity, linguistic exclusion, and problematic data use are partly rooted in the competitive structure of the market, then governance cannot rely solely on exhortations to responsible innovation. It must address the institutional conditions that shape strategic decision-making. In Homann’s terms, ethical governance must aim at changing the rules under which firms pursue innovation, rather than merely expecting them to act against their own competitive interests.

This point is crucial for the later argument of this paper. The debate over closed versus open AI systems often appears as a normative disagreement over transparency, safety, or freedom of access. Yet from an institutional economic perspective, it is also a question of how different governance models interact with cost structures, appropriability, and market concentration. A fully closed regime may stabilise private investment but intensify asymmetries of control and weaken accountability. A fully open regime may broaden access and scrutiny but undermine incentives for some firms while increasing concerns about misuse or uncontrolled diffusion. The relevant governance problem is therefore how to structure a framework that alters these trade-offs in a more balanced way.

For this reason, the next section turns from industry structure to the central ethical challenges associated with LLM development. Having clarified the economic environment in which these systems are produced, the analysis can now examine more precisely how issues of fairness, transparency, and data governance emerge within that environment and why they should be interpreted as institutional rather than purely technical or moral problems.

## Institutional Design Options for Ethical AI Development

### Regulatory Approaches: Aligning Innovation with Social Values

One of the most significant ethical challenges associated with large language models concerns the unequal representation of languages, cultural contexts, and social realities in the processes of model training and deployment. Although large language models are often presented as general-purpose systems, their apparent universality can be misleading. Their performance depends heavily on the availability, accessibility, and economic relevance of the linguistic material on which they are trained. This means that the capabilities of LLMs are not distributed evenly across languages, but are shaped by asymmetries in digital text production, data availability, and commercial value (Brown et al., 2020; Ferdaus et al., 2024).

At a technical level, this problem begins with the basic fact that language models learn from patterns in large-scale corpora. Languages with extensive digitised content, large online user communities, and strong integration into global knowledge and media infrastructures are more likely to be represented in training data at scale. This gives high-resource languages—especially English—a structural advantage. Such languages provide a richer statistical basis for model training, more opportunities for evaluation and refinement, and stronger incentives for commercial optimisation. By contrast, languages with less digital presence, fewer curated datasets, or lower market relevance are more likely to be underrepresented in ways that affect model quality and robustness (Brown et al., 2020).

However, the ethical significance of this asymmetry goes beyond differences in technical performance. Large language models increasingly function as infrastructures for communication, information access, content generation, and knowledge mediation. If these infrastructures systematically perform better in dominant languages than in others, then they risk reinforcing pre-existing inequalities in participation and access. Users operating in linguistically dominant environments are more likely to benefit from accurate, context-sensitive, and functionally rich systems. Users in underrepresented linguistic contexts may instead encounter lower-quality responses, weaker contextual understanding, or reduced applicability in education, administration, and professional settings. The result is not only a technical disparity but a distributional inequality concerning access to the benefits of AI-mediated communication (Ferdaus et al., 2024).

This issue becomes even more serious once language is understood not merely as a formal system of grammar and vocabulary, but as a carrier of cultural meaning, social experience, and historically situated forms of expression. Training corpora do not simply contain neutral linguistic material; they reflect existing patterns of visibility, exclusion, hierarchy, and power. As a result, LLMs may reproduce not only lexical or semantic regularities, but also the uneven distribution of cultural presence found in digital discourse. Dominant languages and discursive traditions are more likely to shape what is treated as normative, legible, and statistically central within a model. Less visible linguistic communities, regional expressions, or culturally specific communicative practices may remain marginal or be represented only in distorted ways (Daly et al., 2021; Ferdaus et al., 2024).

From an ethical point of view, this raises questions of fairness, recognition, and epistemic inclusion. If language models are increasingly used as mediators of knowledge and communication, then unequal linguistic representation is not a peripheral issue. It affects whose perspectives can be articulated effectively, whose communicative practices are treated as intelligible, and whose social worlds become visible within AI systems. In this sense, linguistic inequality intersects with broader concerns about cultural justice. The issue is not only whether a model can technically generate text in many languages, but whether it does so in ways that respect diversity, avoid systematic distortion, and do not privilege commercially dominant linguistic environments as the default standard.

This concern is closely related to wider debates on bias in AI systems. Existing research has repeatedly shown that AI models can replicate and amplify the biases present in their training data, and that such biases are often tied to broader patterns of social exclusion rather than isolated technical errors (Daly et al., 2021; Ferdaus et al., 2024). In the context of LLMs, bias is therefore not exhausted by the presence of offensive stereotypes or discriminatory outputs. It also includes structural forms of exclusion in which certain languages, contexts, or communicative repertoires are less fully represented and therefore less effectively served. A model may appear broadly capable while still reproducing a hierarchically ordered linguistic landscape.

This is precisely where an institutional economic perspective becomes important. The unequal representation of languages in large language models cannot be explained only by technical limitations. It is also shaped by the incentive structure of the market in which such systems are developed. Firms have strong reasons to prioritise languages, user groups, and application contexts that promise the largest returns on investment. Investments in underrepresented languages, minority contexts, or less commercially valuable cultural environments may be ethically important, but they often generate uncertain or limited short-term economic benefits. Under competitive conditions, such investments may therefore be deprioritised in favour of scaling capabilities that are easier to monetise. Linguistic inequality is thus not simply an accidental side effect of innovation; it is partly a consequence of how incentives are distributed in the political economy of AI development (Homann & Suchanek, 2005).

Seen in this light, the problem also illustrates a broader limitation of principle-based AI ethics. Calls for fairness and inclusion remain normatively important, but they do not by themselves explain why exclusionary patterns persist even when such principles are widely endorsed. Homann’s institutional ethics helps clarify that socially desirable outcomes are unlikely to emerge consistently where market incentives do not support them. If building more inclusive multilingual systems entails additional costs without corresponding strategic advantages, then firms cannot be expected to pursue such goals reliably through voluntary self-commitment alone. Ethical concern must therefore be linked to institutional design.

This does not mean that every inequality in model performance can or should be eliminated through regulation. But it does imply that governance debates must take the structural basis of linguistic exclusion seriously. If large language models are becoming part of the infrastructure of public and economic life, then unequal linguistic representation is a question of governance rather than merely an issue of technical optimisation. It concerns the institutional conditions under which inclusion is encouraged, neglected, or rendered economically unattractive. For this reason, linguistic inequality should be treated as a core ethical problem of LLM development and as an important reason to rethink the relationship between commercial incentives, technological design, and public responsibility.

### Transparency Obligations: Shedding Light on Data and Algorithms

A second central ethical challenge of large language models concerns transparency and the closely related problem of accountability. In current debates on AI ethics, transparency is often treated as a key normative requirement because it enables scrutiny, supports trust, and provides a basis for assigning responsibility. In the case of large language models, however, transparency is difficult to achieve and often only partially realised. This is due not only to the technical complexity of such systems, but also to the economic and strategic interests surrounding their development and deployment (OpenAI, 2023; Touvron et al., 2023).

The transparency problem in LLMs operates at several levels. It concerns, first, the training data on which models are built. In many cases, external observers have only limited knowledge of what kinds of data were used, how those data were curated, and according to which criteria material was excluded, filtered, or weighted. Second, opacity concerns the design and training process itself, including architecture choices, optimisation procedures, fine-tuning practices, and safety interventions. Third, the problem extends to deployment contexts, where users often have only limited insight into how models are integrated into products, how outputs are monitored, or what institutional safeguards exist around their use. The result is not merely a lack of technical explanation, but a broader deficit of intelligibility regarding how these systems are produced and governed (OpenAI, 2023; Patidar et al., 2024).

This lack of transparency is ethically significant because it weakens the conditions under which accountability can be meaningfully exercised. Accountability presupposes that relevant actors can be identified, that the basis of decisions or outputs can be examined, and that harmful effects can be traced back to design choices, data practices, or deployment arrangements. Where these conditions are absent, responsibility becomes diffuse. It becomes harder for regulators, researchers, and affected publics to assess whether a model systematically disadvantages certain groups, reproduces problematic assumptions, or relies on questionable forms of data processing. In practice, opacity can therefore shield both technical limitations and governance failures from meaningful external criticism (Ferdaus et al., 2024; Ford, 2021; Widayanti & Mariyanti, 2023).

The importance of this issue becomes especially clear when large language models are used in contexts with social or economic consequences. In such settings, users and institutions may rely on model outputs without being able to assess the basis on which those outputs were generated. Even where LLMs are formally described as assistive tools, their integration into administrative, educational, communicative, or professional environments can create practical dependencies. Ethical concerns about transparency are therefore not reducible to abstract preferences for openness. They concern the possibility of contestation, oversight, and justified trust in systems that increasingly shape access to information and mediate social interaction (Anang et al., 2024; Horváth, 2022).

At the same time, transparency cannot be treated as a simple all-or-nothing requirement. Large language models are complex socio-technical systems, and complete transparency may be neither technically feasible nor normatively sufficient in itself. The relevant question is therefore not whether every element of a model can be made fully transparent, but which forms of disclosure are necessary to enable meaningful accountability. This includes, for example, information about the sources and limitations of training data, documentation of system capabilities and constraints, reporting on evaluation practices, and institutional clarity about who is responsible for deployment decisions and downstream harms. Transparency, in this sense, is not identical with total openness. It is better understood as a structured condition for scrutiny and responsibility (Patidar et al., 2024; Ferdaus et al., 2024).

This distinction is important because the opacity of LLMs is not only the result of technical scale. It is also bound up with proprietary strategies and the logic of market competition. Firms developing large language models often have strong incentives to withhold technical and organisational information because such information constitutes a valuable strategic asset. Model weights, training procedures, safety methods, and data pipelines may be regarded as commercially sensitive because they affect competitive advantage, product differentiation, and future revenue opportunities. Under such conditions, opacity can become economically rational even when it is ethically problematic (Langenkamp & Yue, 2022; Khanjani & Sulaiman, 2011).

From the perspective of institutional economic ethics, this is decisive. If greater transparency creates costs while secrecy protects strategic interests, then it is unrealistic to expect firms to provide far-reaching disclosure voluntarily and consistently across the sector. Appeals to ethical responsibility may lead to selective self-regulation, but they do not alter the basic incentive structure under which opacity remains advantageous. Homann’s framework helps clarify that transparency deficits in AI development are therefore not merely failures of ethical awareness. They are structurally linked to a market environment that rewards control over information and does not systematically compensate firms for the public value of openness (Homann & Suchanek, 2005).

This point also helps explain why transparency debates in AI often remain unsatisfactory when they are framed only in normative terms. It is not enough to state that transparency is desirable. The more difficult question is how governance arrangements can be designed so that relevant forms of transparency become compatible with innovation and competition. This may require differentiated obligations rather than undirected calls for openness. For example, institutional arrangements could distinguish between public disclosure, regulator access, third-party auditing, and controlled research access. Such distinctions matter because the ethical problem is not simply whether information is hidden, but how the institutional design of disclosure affects accountability, safety, and competitive incentives (Petit & De Cooman, 2020; Ford, 2021; Ayinla et al., 2024).

Seen in this way, the problem of transparency leads directly to the broader governance question of the paper. The ethical challenge is not solved by choosing either complete openness or complete secrecy. Rather, it requires a more differentiated approach that identifies which layers of large language models should be subject to what forms of access, by whom, and for what purposes. This is one of the reasons why the concept of partially open AI governance later becomes central to the argument. Transparency is ethically indispensable, but it must be institutionally specified if it is to function under real-world competitive conditions.

### Data Governance and Intellectual Property

A third major ethical challenge in the development of large language models concerns data governance and intellectual property. Large language models depend on vast quantities of textual material in order to acquire linguistic competence, contextual flexibility, and broad generalisation capacity. Yet the collection, processing, and reuse of such data raise difficult questions regarding provenance, ownership, legitimacy, and control. These issues are ethically significant not only because they concern legal rights, but also because they reveal how value is extracted from communicative and cultural resources under conditions of limited transparency and asymmetric power (Daly et al., 2021; von Engelhardt, 2008).

At the centre of the problem lies the fact that the datasets used to train large language models are often assembled from heterogeneous digital sources whose normative status is not uniform. Publicly accessible text may include copyrighted material, user-generated content, forum discussions, educational resources, journalistic writing, or professionally authored works. The mere availability of such material in digital form does not in itself settle the question of whether its large-scale computational reuse is ethically justified. Even where legal doctrines permit certain forms of data processing, broader concerns remain regarding authorship, consent, recognition, and the fairness of deriving economic value from resources created by others without meaningful reciprocity (von Engelhardt, 2008; Ford, 2021; Ayinla et al., 2024; Yang & Lee, 2024; Wang, 2024).

This issue is particularly important because the economic success of large language models depends in part on the quality and scale of the data to which developers have access. Data is not simply an input among others; it is one of the core resources through which model performance and commercial value are generated. The incentive to acquire and process ever larger volumes of textual material is therefore strong, especially in highly competitive markets where scale and capability are closely connected. Under such conditions, firms may be encouraged to adopt expansive data practices even where the legal or ethical status of particular sources remains unclear. Questions of intellectual property are thus not external constraints on AI development; they are internal to the political economy of model training.

The ethical problem is not exhausted by formal ownership claims. It also concerns the legitimacy of appropriation. Language models derive their capabilities from corpora that reflect collective social labour, cultural production, and public communication. Much of the material on which models are trained has been produced without any expectation that it would be incorporated into commercial AI systems. This creates a tension between the technological and economic value generated by model developers and the often diffuse, unrecognised, or uncompensated contributions of the individuals and communities whose textual production makes such systems possible. In this sense, questions of data governance are also questions of distributive fairness and recognition.

A further difficulty is that weak data governance can undermine the ethical assessment of models more broadly. If the provenance and composition of training datasets are insufficiently documented, then it becomes harder to evaluate potential biases, exclusions, and representational distortions. Data governance is therefore closely tied to transparency. Without information about what kinds of data were included, how those data were curated, and according to which standards material was selected or filtered, external scrutiny remains limited. In practice, poor data governance can reinforce opacity and weaken accountability at the same time (Daly et al., 2021; Ferdaus et al., 2024).

This issue also intersects with transaction costs and regulatory uncertainty. In rapidly evolving technological fields, legal standards may lag behind technical practice, and the costs of fully verifying data provenance or obtaining permissions at scale may be substantial. From an economic standpoint, firms may therefore face incentives to proceed under legal ambiguity rather than incur the costs of stricter governance in advance. Where enforcement is limited or sanctions uncertain, strategies of aggressive data accumulation may become economically rational even if they remain ethically contested. This helps explain why problematic data practices can persist even in firms that publicly endorse responsible AI principles (von Engelhardt, 2008; Petit & De Cooman, 2020).

From the perspective of institutional economic ethics, this pattern is highly significant. Homann’s framework suggests that ethically desirable data practices cannot be expected to stabilise purely through voluntary commitment if they impose additional costs that competitors can avoid. More careful documentation, stricter provenance standards, or more restrictive use of contested data may all be normatively desirable, but they may also slow model development, reduce available training material, or create competitive disadvantages in a market driven by speed and scale. In such a setting, ethical deficits in data governance are better understood as structurally induced outcomes than as isolated failures of goodwill (Homann & Suchanek, 2005).

This does not imply that legal regulation alone will resolve all tensions surrounding data governance and intellectual property. It does imply, however, that the governance of large language models must address these issues institutionally rather than merely morally. If the current market order rewards extensive data extraction while leaving the costs of ethical uncertainty diffuse or externalised, then problematic practices are likely to continue. Governance must therefore ask how incentives can be restructured so that more responsible forms of data use do not remain systematically disadvantageous.

For the purposes of this paper, this point is especially important because it reinforces the broader claim that ethical challenges in LLM development are interconnected. Data governance is not a separate legal appendix to the debate over language models. It is linked to transparency, accountability, and the distribution of benefits and burdens within AI ecosystems. The question of who may access, use, document, and benefit from data is central to the institutional design of AI development. This is why the subsequent discussion of open, closed, and partially open systems must also be understood as a debate about how data-related rights, duties, and forms of access ought to be governed.

### Future Perspectives: How (Business) Ethics Can Foster Fairness in AI

The question of fairness in AI is not just a technical challenge—it is fundamentally a business ethics issue. As we have discussed, language models and other AI systems are increasingly influencing decisions and mediating information in society. Ensuring these systems are fair (unbiased and just in their outcomes) and transparent is critical to prevent AI from exacerbating social inequalities. Business ethics provides both a framework for understanding this issue and a toolkit for addressing it, especially when combined with forward-looking institutional design.

Drawing on Homann’s institutional economics, one clear perspective emerges: enduring fairness in AI will only be achieved if the economic incentives are aligned with ethical outcomes. Companies developing AI need to find competitive advantage in doing the right thing, not see ethics as merely a cost or constraint. This suggests that *the market environment itself must be engineered* to reward fairness. Some ideas for the future involve creating what we might call ethical competitive advantages. For instance, suppose regulators and industry together create a widely recognized “AI Fairness” certification or seal. Consumers (whether individuals or enterprise clients) might then favor certified products, and being certified could become a selling point. Over time, this market-driven reward for fairness would push firms to seek certification as part of their business strategy, effectively internalizing the cost of fairness efforts as necessary R&D. Similarly, if governments give procurement preference or tax incentives to companies with demonstrably fair AI, this alters the economic calculus in favor of fairness.

Another perspective is the potential role of industry self-regulation in a pre-competitive stage. Leading AI firms could collaborate on creating shared resources that improve fairness across the board—such as large diversified training datasets or bias mitigation libraries—because it benefits the industry’s social license to operate if AI is seen as fair. This kind of cooperation is not unheard of; in other domains, firms have joined forces on safety standards knowing that one big accident can tarnish all players (consider how nuclear power companies collectively have industry safety bodies). If the major players agree on certain fairness standards or tools, and perhaps even pool data to tackle bias (in a privacy-conscious way), they might reduce the risk of reputational disasters that could invite heavy regulation. In essence, enlightened self-interest might lead to a form of *ethical cartelization*, where companies compete on usual features but “collude” to ensure a baseline of ethical practices for the good of all.

However, history shows that relying on voluntary action alone is risky—regulation and public oversight are typically needed to sustain high standards. Thus, a hybrid model appears most promising. We have already touched on elements of this: a mix of regulation, market incentives, and stakeholder participation. Envision a scenario 5–10 years from now where the AI landscape is governed by a coordinated system: international guidelines set broad principles (much like human rights treaties do), national laws enforce core requirements (transparency, auditability, accountability for outcomes), industries have developed ethics labels and audit regimes, and civil society plus affected communities have formal channels to influence AI services (e.g., an AI ombudsperson or citizen panels feeding into periodic policy revisions).

Concretely, such a hybrid system might include innovations like a publicly funded “Fair AI” consortium that regularly evaluates major AI models for bias and reports to the public (similar to how consumer reports test products) If a model consistently ranks poorly, it would face pressure to improve or risk loss of business and credibility. Another innovation could be an AI ethics sandbox: a controlled environment where companies can test new kinds of models or uses with enhanced monitoring and input from ethicists and community representatives, allowing learning and adjustment before wider release. This would encourage innovation in areas that might be sensitive, by giving companies a pathway to develop them responsibly rather than avoiding them altogether or deploying recklessly.

In the specific context of *partially open-source approaches*, the future might see more blending of open and closed models to harness collaborative improvement while maintaining safety and incentives. One could imagine a regime where foundational models (like fundamental language understanding models) are maintained as open infrastructure by a consortium (possibly with public funding), upon which companies build proprietary fine-tuned systems for specific tasks. In this model, fairness and inclusivity could be managed at the base level (the open foundation model trained on globally diverse data, overseen by an international body), while companies compete in providing value-added services. This partially open ecosystem might mitigate the concentration of power, as no single company controls the core technology, and it spreads the benefits of AI development more broadly (Langenkamp & Yue, 2022 discuss how open-source ML can democratize innovation).

Finally, education and culture will play a role in future progress. As awareness grows that AI can discriminate or be unfair, stakeholders from investors to employees are increasingly pushing companies to take ethics seriously. Ethical AI is becoming part of corporate social responsibility. Just as sustainability and carbon footprint are now boardroom issues due to shareholder and public pressure, we can expect AI ethics (fairness, transparency, impact on society) to become a standard agenda item for corporate governance in tech and beyond. This cultural shift, where doing unethical AI is as frowned upon as dumping toxic waste, will reinforce all the institutional measures we have discussed.

### Ethical Challenges as Governance Challenges

Taken together, the preceding analysis shows that the ethical challenges of large language models are not isolated or merely technical problems. Linguistic inequality, opacity, and contested data use are closely interconnected and arise within the same structural environment. Each of these problem areas reflects a tension between socially desirable objectives and the incentive structure of a rapidly expanding market for AI systems. Firms are encouraged to prioritise scale, speed, and strategic control, while the costs of inclusion, transparency, and careful data governance are often internalised only weakly or unevenly. Ethical deficits therefore persist not simply because relevant actors lack awareness of normative concerns, but because the institutional setting does not reliably reward their mitigation (Homann & Suchanek, 2005).

This point is crucial for the argument of the paper. If the core ethical problems of LLM development are structurally embedded, then they cannot be addressed adequately through moral appeals alone. Principle-based calls for fairness, accountability, and responsibility remain important, but they do not by themselves alter the competitive environment in which firms make strategic decisions. An institutional perspective therefore requires a shift from the formulation of abstract ethical demands to the design of governance arrangements capable of reshaping incentives in practice (Homann & Blome-Drees, 1992; Pies, 2011).

At the same time, the preceding discussion also indicates that these ethical challenges are not all of the same kind. Some concerns relate primarily to disclosure and external scrutiny, while others concern access, representation, control, or the distribution of economic value. This suggests that governance cannot be reduced to a single principle or a simple regulatory formula. In particular, it raises doubts about whether the opposition between “open” and “closed” AI systems is sufficient to capture the complexity of the ethical and economic trade-offs involved. Full openness may improve transparency and participation in some respects, but it may also create new risks and undermine certain investment incentives. Strict closure may protect strategic assets and facilitate control, but it can also deepen opacity, weaken accountability, and reinforce concentration.

For this reason, the next section turns to the governance debate surrounding open and closed AI models. The aim is not to resolve that debate by endorsing one of its poles in an abstract way, but to examine how different degrees of openness affect the ethical challenges discussed above. This step is necessary in order to develop the central proposal of the paper: a model of partially open AI governance that seeks to balance transparency, innovation, accountability, and public responsibility under real-world institutional conditions.

## Open, Closed, and Partially Open AI Systems

### The Governance Relevance of Openness in Large Language Models

The question of whether large language models should be developed as open or closed systems has become one of the most important controversies in contemporary AI governance. At first glance, the issue appears straightforward: closed systems seem to favour control, investment protection, and risk management, whereas open systems appear to promote transparency, participation, and collaborative innovation. Yet this contrast is misleading if it is treated as an exhaustive conceptual distinction. In practice, the governance of large language models is not determined by a single choice between total secrecy and unrestricted openness. Rather, different components of these systems are disclosed, withheld, licensed, or documented in different ways, and these choices have distinct ethical and economic consequences (Langenkamp & Yue, 2022; Baiasu, 2020).

This matters because openness is not merely a technical design feature. It is a governance variable. Decisions about whether model weights are accessible, whether training data are documented, whether architectures are published, or whether third parties can meaningfully audit model behaviour shape the institutional environment in which AI systems are developed and evaluated. They affect who can scrutinise a system, who can improve or adapt it, who can benefit economically from it, and who bears the risks associated with its deployment. In that sense, openness structures the distribution of power, accountability, and innovation capacity across AI ecosystems.

For this reason, the debate over open and closed AI systems should not be understood primarily as a cultural conflict between “open” and “proprietary” communities. It is more accurately interpreted as a conflict over institutional design in a field characterised by high development costs, strong strategic incentives, and socially significant externalities. The ethically relevant question is therefore not whether openness is desirable in the abstract, but which forms of openness support public accountability, democratic control, and socially beneficial innovation without simply dissolving the conditions under which model development takes place (Bogiatzis-Gibbons, 2024; Homann & Suchanek, 2005).

From the perspective of institutional economic ethics, this issue is especially important because it concerns the relationship between moral demands and incentive structures. Calls for transparency and openness are normatively persuasive, but they remain incomplete if they do not specify how these demands interact with competition, appropriability, and risk. The governance challenge lies precisely in developing arrangements that do not assume that firms will sacrifice strategically valuable assets simply because openness is ethically attractive. Instead, the relevant task is to design institutions under which different forms of openness become compatible with the broader organisation of incentives in AI development (Homann & Blome-Drees, 1992; Pies, 2011).

### Closed AI Systems: Control, Appropriability, and Strategic Secrecy

Closed AI systems are often defended on the grounds that they are necessary for sustaining innovation under competitive market conditions. Advanced large language models require substantial investments in computing infrastructure, engineering capacity, data acquisition, and model refinement. In such a setting, firms have strong reasons to protect the technical and organisational assets that generate competitive advantage. Closed development models allow firms to retain control over weights, training pipelines, evaluation systems, and commercial deployment strategies. From an economic perspective, this can be understood as a classic appropriability problem: if innovation is too easily replicable, private incentives to undertake costly model development may weaken (Agrawal et al., 2019, 2022).

This argument has considerable force in the case of foundation models and large language models. Unlike many earlier digital goods, advanced LLMs depend not only on code but on large-scale, expensive, and often highly integrated infrastructures. The firms that finance these systems may therefore regard secrecy as a rational means of preserving expected returns. In addition, closed models can support strategic product integration. Companies that control both a model and the ecosystem into which it is deployed may use that control to consolidate market position, shape standards, and expand dependence on their infrastructure. Closure is thus not merely a passive withholding of information; it can be part of an active market strategy aimed at technological leadership and ecosystem control (OpenAI, 2023; Acemoglu & Restrepo, 2020).

A second argument in favour of closure concerns safety and misuse. Developers may claim that restricting access to model internals, weights, or full technical capabilities is necessary in order to prevent harmful downstream applications, uncontrolled replication, or the irresponsible deployment of systems in sensitive contexts. This argument has become more prominent as large language models are seen not only as commercially valuable but also as potentially capable of facilitating disinformation, manipulation, or other forms of scalable harm. From this standpoint, closure is defended as a mechanism of responsible stewardship rather than merely as a means of commercial protection (OpenAI, 2023; Abiri, 2024).

However, these arguments do not settle the ethical question. Closed systems also produce substantial normative costs. Most importantly, they can intensify opacity and thereby undermine the conditions of accountability. If researchers, regulators, or affected publics do not know what kinds of data were used, how systems were evaluated, or what safeguards were implemented, then independent scrutiny becomes severely limited. This is ethically problematic not only because it restricts transparency, but because it concentrates epistemic authority in the hands of the developer. The more central LLMs become in infrastructures of communication, knowledge access, and decision support, the more problematic it is if the basis of their operation remains substantially shielded from public examination (Ferdaus et al., 2024; Bogiatzis-Gibbons, 2024).

Closed systems also raise concerns regarding democratic legitimacy. Where AI models are deployed at scale and begin to shape communicative environments, public institutions, or market relations, governance cannot be left entirely to private actors who control access to information about those systems. Bogiatzis-Gibbons (2024) is therefore right to emphasise that AI governance must not be reduced to individual accountability alone. Institutional arrangements matter because AI systems increasingly affect public life in ways that demand forms of control exceeding corporate self-regulation. Closure may protect strategic interests, but it can also weaken the social and democratic conditions under which those interests remain publicly contestable.

For this reason, closed models cannot simply be justified by pointing to innovation incentives or safety risks. They must also be evaluated in terms of how much opacity a legitimate AI order can tolerate. Once this question is raised, it becomes clear that the appeal of closure is real but incomplete. It identifies an important problem—namely the need for control, appropriability, and managed deployment—but it does not by itself provide a sufficient governance model for ethically defensible AI development.

### Open AI Systems: Transparency, Participation, and Their Limits

Open AI systems are often presented as a corrective to the deficits of proprietary control. Openness is associated with transparency, reproducibility, decentralised innovation, and broader access to technological capabilities. In the context of large language models, more open arrangements may allow independent researchers to investigate system behaviour, identify harmful biases, evaluate claims made by developers, and adapt models to contexts that are neglected by dominant commercial actors. Open approaches may therefore contribute to a more plural and participatory AI ecosystem, especially where corporate concentration would otherwise limit access to advanced systems (Langenkamp & Yue, 2022; Touvron et al., 2023).

This promise is ethically significant. If large language models are becoming general-purpose infrastructures, then broader access may serve as a counterweight to concentration of power. Open systems can support experimentation by academic institutions, smaller firms, and civil-society actors that would otherwise remain dependent on a small number of model providers. In addition, openness may improve accountability by making at least some components of a system available for external scrutiny. This can be especially important in relation to the ethical concerns discussed earlier in the paper, such as linguistic inequality, opacity, and problematic data governance. More open systems may make it easier to identify exclusionary patterns, develop alternative use cases, and challenge the informational asymmetries maintained by dominant firms (Bender et al., 2021; Ferdaus et al., 2024).

Openness may also have democratic advantages. If the control of AI capabilities is concentrated in a small number of private firms, then public debate about the direction of AI development is structurally constrained. More open systems can widen the field of actors able to participate meaningfully in AI research, critique, and adaptation. In this respect, openness is not merely a technical principle but a question of access to social and epistemic power. Comparable questions arise wherever openly available information becomes a resource. As Baiasu (2020) argues for open-source information more generally, accessibility alone does not settle the ethical status of its use, which remains bound to questions of provenance, responsibility, and legitimate purpose. The same asymmetry applies to open AI systems. Availability and accountability are not the same thing.

At the same time, the ethical and political appeal of openness should not be romanticised. One important reason is conceptual imprecision. The term “open” is often used loosely in AI discourse, even though systems labelled as open may differ substantially with regard to architecture disclosure, weight access, training data transparency, licensing conditions, documentation quality, or downstream use restrictions. A model may be called open while critical information remains unavailable. The rhetoric of openness can therefore create a misleading impression of transparency and accessibility where only some components are meaningfully disclosed (Touvron et al., 2023; Langenkamp & Yue, 2022).

A second limitation concerns risk. Openness can facilitate beneficial experimentation, but it may also lower barriers for harmful or irresponsible use. Models that are widely accessible may be modified, redeployed, or integrated into applications without adequate safeguards. This concern is often raised in relation to dual-use risks, unsafe deployment, and the difficulty of controlling downstream applications once a model becomes widely available. The ethical challenge here is not merely whether openness is good, but how to govern the conditions under which openness creates public value without enabling forms of harm that are difficult to reverse (Abiri, 2024; OpenAI, 2023).

Third, openness may create tensions with private investment incentives. From a market perspective, full openness can reduce the ability of firms to appropriate returns from costly model development. This does not mean that open innovation lacks economic value. But it does mean that the relationship between openness and innovation is institutionally mediated rather than self-evident. Some forms of openness may stimulate follow-on innovation while others may reduce willingness to bear the up-front costs of developing frontier systems. Agrawal et al. (2019, 2022) help illuminate this point by showing that AI development must be understood in relation to incentive structures, prediction value, and organisational adaptation. What matters is not openness alone, but how openness interacts with the economics of AI production.

These considerations suggest that open AI systems are ethically significant but not normatively self-sufficient. They address real deficits of proprietary concentration and opacity, yet they also raise unresolved questions of safety, institutional control, and innovation incentives. The task is therefore not to idealise openness as the obvious ethical answer, but to specify more carefully which dimensions of openness matter for which governance purposes.

### Why the Binary Opposition Between Open and Closed Is Inadequate

Once the arguments on both sides are examined more carefully, the standard contrast between open and closed systems appears too coarse to capture the governance problem adequately. In practice, AI systems are rarely fully open or fully closed in every relevant respect. Instead, they consist of multiple components that can be governed differently. A system may disclose architecture while withholding weights. It may release weights but not training data. It may provide extensive documentation but restrict commercial reuse. It may allow external testing while preventing unrestricted deployment. The governance question is therefore layered and multidimensional rather than binary (Langenkamp & Yue, 2022; Baiasu, 2020).

This point is crucial because many ethical disagreements in AI governance are in fact disagreements about which dimensions of openness matter most. Researchers concerned with bias and accountability may prioritise data transparency and evaluation access. Firms concerned with appropriability may focus on protecting weights and deployment pipelines. Public actors concerned with democratic control may demand institutional forms of access for regulators or auditors rather than indiscriminate public release. Safety-oriented arguments may support disclosure in some respects and restriction in others. The binary vocabulary of open versus closed obscures these distinctions and thereby weakens analytical precision (Bogiatzis-Gibbons, 2024; Abiri, 2024).

The inadequacy of the binary distinction is also apparent from an institutional economic perspective. Different forms of openness affect incentives differently. Public release of technical documentation may have very different economic consequences from unrestricted release of model weights. Limited access for auditors may be compatible with commercial incentives in a way that full public disclosure is not. Similarly, data transparency may improve accountability while requiring forms of governance that differ from those appropriate for source code or deployment rights. Once these distinctions are recognised, it becomes clear that openness is not a single policy variable. It is a bundle of possible governance choices, each with distinct effects on competition, accountability, and control (Homann & Suchanek, 2005; Pies, 2011).

This is precisely where the earlier formulation of “partially open-source” needed sharpening. If partial openness is not specified more carefully, it risks sounding like an intuitive compromise rather than a theoretically grounded concept. Partial openness should not mean mere incompleteness of disclosure. It should refer to a principled differentiation of access regimes across distinct components of an AI system, justified by reference to ethical and economic considerations.

### Partially Open AI Systems as a Structured Governance Concept

A partially open AI system can therefore be defined as a system in which different components are made accessible under differentiated institutional conditions rather than under a single logic of full public openness or full proprietary closure. Such a system is not simply “half open.” It is governed through a layered access structure that distinguishes among what is disclosed, to whom, under what conditions, and for what purposes. The central idea is that openness should be specified functionally rather than rhetorically.

For the purposes of large language models, at least five dimensions of openness are especially relevant. First, there is architectural openness, meaning disclosure of the general structure and technical design of the model. Second, there is weight openness, referring to access to the trained parameters that make practical reuse and adaptation possible. Third, there is data openness, which includes information about training data sources, provenance, composition, and curation practices. Fourth, there is documentation and evaluation openness, covering model cards, known limitations, testing results, and safety procedures. Fifth, there is deployment openness, referring to the terms under which third parties may access, fine-tune, integrate, or redistribute the model. These dimensions can vary independently, and each has different implications for accountability, competition, and risk.

This more precise formulation has several advantages. First, it makes the governance debate analytically tractable. Instead of arguing abstractly about whether a model is open, one can ask which kinds of openness are normatively necessary and institutionally feasible. Second, it allows for targeted responses to specific ethical concerns. If the primary problem is weak accountability, then documentation, evaluation, and controlled audit access may be more important than unrestricted release of all model assets. If the concern is concentration of power, then broader access to certain model components may be justified even where some restrictions remain. Third, differentiated openness can accommodate the fact that innovation incentives and public accountability do not always point in the same direction.

This is the point at which the connection to Homann’s institutional economic ethics becomes most productive. A partially open governance model does not assume that firms will voluntarily choose ethically preferable arrangements out of moral conviction alone. Instead, it asks how institutions can be designed so that forms of openness relevant to accountability and fairness are introduced without collapsing the incentive structure that sustains investment and development. In other words, partial openness is attractive precisely because it treats AI ethics as a problem of rule design under conditions of competition. It seeks to transform the governance environment rather than merely criticise firm behaviour from the outside (Homann & Blome-Drees, 1992; Homann & Suchanek, 2005).

At the same time, partial openness also has democratic significance. If AI systems are to remain subject to meaningful public scrutiny and contestation, governance must create structured pathways through which relevant actors can gain access to information and exert oversight. This does not necessarily require unrestricted openness for everyone in every respect. But it does require more than voluntary opacity moderated by occasional corporate disclosure. In this sense, partially open governance can also be understood as an institutional response to the problem of democratic control raised in recent debates on AI governance (Bogiatzis-Gibbons, 2024; Abiri, 2024).

For these reasons, partially open AI systems should not be seen as an ad hoc compromise between open and closed models. Properly understood, they represent a more realistic governance architecture for a domain in which full openness and full closure each capture only part of the ethical and economic problem. The following section therefore develops this idea into a more explicit governance proposal for large language models.

## A Partially Open Governance Model for Large Language Models

### From Conceptual Distinction to Governance Design

The previous section argued that the opposition between open and closed AI systems is too crude to capture the ethical and institutional challenges associated with large language models. If this diagnosis is correct, the next step must be to move from conceptual clarification to governance design. The question is no longer whether openness is desirable in the abstract, but how differentiated forms of openness can be institutionalised in ways that respond to accountability deficits, unequal access, safety concerns, and innovation incentives simultaneously.

This shift is important because governance debates around large language models often oscillate between two unsatisfactory poles. On one side, there is the expectation that firms should simply disclose more and act more responsibly. On the other side, there is the assumption that strong proprietary control is unavoidable because frontier AI systems are expensive, strategically valuable, and potentially risky. Neither position is sufficient on its own. A governance model for LLMs must take seriously both the normative demand for transparency and the economic structure of AI development, including the role of appropriability, strategic rivalry, and industrial organisation (Varian, 2018; Agrawal et al., 2019).

A partially open governance model is proposed here as a way of addressing this tension institutionally. Its basic premise is that openness should not be distributed uniformly across all components of a large language model system. Instead, access rights, disclosure obligations, and oversight mechanisms should be differentiated according to the governance function of each component. Such a model does not treat openness as a symbolic value in itself, but as a practical instrument for structuring accountability, participation, and innovation under real-world market conditions (Bommasani et al., 2021; Zhang et al., 2023; Zhang Z., 2024).

From the perspective of institutional economic ethics, this approach has a clear rationale. Homann’s central insight is that ethical objectives must be embedded in rules that alter incentives rather than left to voluntary self-restraint alone (Homann & Suchanek, 2005). Applied to large language models, this means that governance should not assume that firms will consistently choose costly transparency, careful data governance, or broad linguistic inclusion in the absence of institutional support or pressure. A partially open governance model seeks to create an order in which at least some ethically relevant forms of openness are stabilised through institutional arrangements rather than through discretionary goodwill.

### Differentiated Layers of Openness

A workable governance model for large language models should distinguish at least five layers of openness, each of which raises different ethical and economic questions.

The first layer concerns architectural transparency. This includes disclosure of the general structure of a model, broad information about training objectives, and the overall technical design. Such disclosure can support scientific understanding and public debate without necessarily requiring firms to surrender all competitively relevant details. In many cases, architectural transparency imposes lower strategic costs than full release of model parameters while still improving intelligibility and comparability across systems (Bommasani et al., 2021; OpenAI, 2023).

The second layer concerns documentation and evaluation transparency. This includes model cards, information about known limitations, evaluation benchmarks, safety tests, intended use conditions, and forms of post-deployment monitoring. This layer is crucial for accountability because it allows external actors to assess what claims developers make about model capabilities and risks. It also helps shift AI governance away from vague ethical branding toward more concrete and reviewable disclosures. Informed regulatory approaches increasingly stress that such evaluative transparency is essential if governance is to move beyond broad principle statements toward operational accountability (Chun & Elkins, 2024; Berengueres, 2024).

The third layer concerns data governance transparency. Full public release of all training data may be neither feasible nor normatively desirable, especially where privacy, copyright, and contractual restrictions are involved. But this does not justify complete opacity. At a minimum, a partially open governance model should require structured disclosure regarding data provenance, categories of sources, filtering procedures, and major limitations of the dataset composition. Such transparency is necessary if questions of fairness, bias, and legitimacy are to be assessed in a meaningful way (Daly et al., 2021; Bommasani et al., 2021).

The fourth layer concerns controlled weight and model access. This is perhaps the most contentious dimension because model weights often embody a significant share of the economic value of a system. Full public release may support adaptation and decentralised innovation, but it may also create appropriation concerns and increase downstream risk. A partially open governance model therefore allows for differentiated access regimes. For example, access to weights might be granted under specific research, regulatory, or licensed conditions rather than through unrestricted public release. This kind of arrangement reflects the idea that meaningful oversight does not always require universal openness, but it does require more than unilateral corporate discretion (Langenkamp & Yue, 2022; Touvron et al., 2023).

The fifth layer concerns deployment governance. Even where some technical components remain restricted, governance can require transparency about deployment domains, prohibited uses, auditing obligations, and institutional safeguards around implementation. This is particularly important because many harms associated with large language models emerge not simply from the existence of the model, but from the contexts in which it is integrated and relied upon. Public-facing governance frameworks therefore need to regulate not only the model as an artefact, but also the terms under which it enters socially consequential domains (Manias et al., 2023; Cuéllar & Huq, 2022).

This layered approach has a significant advantage: it disaggregates the governance problem. Instead of demanding either full openness or broad secrecy, it allows policy and institutional design to respond to the specific functions of different model components. That makes it better suited to balancing the competing concerns identified throughout this paper.

### Incentive Compatibility and the Economic Logic of Partial Openness

The normative attractiveness of partial openness depends on whether it can be made incentive-compatible. From an institutional economic point of view, this is decisive. A governance proposal that ignores the strategic environment of firms may be ethically appealing in theory while remaining unstable in practice. The key question is therefore whether differentiated openness can be structured in a way that preserves sufficient incentives for investment and development while reducing the ethical deficits associated with opacity, concentration, and weak accountability.

Here the economics of artificial intelligence become especially relevant. AI systems generate value not only because of technical novelty, but because they function as prediction and decision-support technologies embedded in larger organisational and market structures (Agrawal et al., 2022). Their development is shaped by industrial organisation, platform effects, and strategic control over complementary assets such as infrastructure, data access, and distribution channels (Varian, 2018; Acemoglu & Restrepo, 2020). This means that the economic consequences of openness vary depending on which layer is opened and which assets remain proprietary.

A partially open governance model can therefore be justified economically if it distinguishes between what firms must be able to appropriate and what society must be able to scrutinise. For instance, requiring stronger documentation, more structured evaluation disclosure, or regulator access to certain forms of information may impose lower economic costs than forcing unrestricted release of all model weights. Similarly, controlled access arrangements can allow public-interest oversight or scientific evaluation without completely removing the possibility of commercial return. The aim is not to eliminate strategic advantage altogether, but to prevent its pursuit from systematically undermining accountability and public legitimacy (Homann & Suchanek, 2005; Agrawal et al., 2019).

This logic is consistent with Homann’s broader argument that ethically desirable outcomes must be embedded in an order that actors can rationally follow. If transparency obligations, audit requirements, or differentiated disclosure rules are designed in a way that is predictable and generalisable across competitors, then individual firms are less likely to be disadvantaged merely because they comply. In this respect, a partially open governance regime can reduce the dilemma structure identified earlier in the paper: it can help ensure that firms do not bear ethical costs unilaterally while competitors free-ride on opacity or weak governance.

The same logic applies to fairness-related concerns. Measures that improve accountability for bias, linguistic exclusion, or harmful model behaviour often require access to information, evaluation interfaces, or documentation rather than unrestricted public disclosure of everything. This suggests that better governance does not always depend on maximal openness. In some cases, targeted openness combined with institutional oversight may generate a more stable balance between safety, fairness, and innovation than either extreme model.

### Partial Openness, Democratic Control, and Public Legitimacy

A further advantage of partially open governance is that it can strengthen democratic control without collapsing into either technocratic opacity or unrestricted release. Recent scholarship has rightly emphasised that AI governance should not be framed solely in terms of individual responsibility or corporate ethics. Because AI systems increasingly shape public communication, administrative functions, and access to knowledge, questions of governance are also questions of democratic legitimacy (Bogiatzis-Gibbons, 2024; Cuéllar & Huq, 2022).

From this perspective, the problem with strongly closed AI systems is not only that they are insufficiently transparent, but that they place too much authority over socially consequential infrastructures in the hands of a small number of private actors. At the same time, a purely libertarian conception of openness may also be inadequate, because democratic governance requires not only access but institutionally organised responsibility. Public legitimacy depends on the existence of pathways for scrutiny, contestation, and oversight. These pathways can take different forms, including public reporting obligations, access rights for regulators, controlled research access, or governance arrangements that require disclosure of relevant limitations and harms (Manias et al., 2023; Chun & Elkins, 2024).

A partially open governance model supports these goals by creating differentiated channels of access rather than reducing governance to a simple public/private dichotomy. It allows the question of “who gets access to what” to be answered institutionally. Regulators may require one level of access; independent auditors another; the public a third. Such differentiation is not a retreat from openness but a recognition that governance must specify audiences, rights, and purposes. This is especially important in contexts where unrestricted openness would not automatically yield meaningful accountability, either because the information would be unusable to most actors or because the relevant issue is not raw access but institutional capacity to interpret and act upon that access.

This also connects to broader debates about alignment and human values. Christian (2020) has argued that the alignment problem is not reducible to engineering in a narrow sense; it concerns the social and normative conditions under which machine learning systems are oriented toward humanly valuable ends. A similar insight applies here. The governance of large language models is not simply about choosing a preferred technical architecture of openness. It is about designing institutions that make it more likely that these systems remain responsive to public values, contestable by affected actors, and constrained by forms of accountability that exceed firm-level discretion (Christian, 2020).

### Partial Openness as an Institutional Response

The central claim of this paper is therefore not that partial openness is a perfect solution. Rather, it is that it provides a more realistic institutional response to the ethical and economic tensions of LLM governance than either full closure or fully undifferentiated openness. It recognises that frontier AI systems are produced in competitive environments structured by strong incentives for secrecy, appropriation, and strategic control. But it also insists that these incentives cannot be treated as sufficient grounds for maintaining broad opacity where public accountability and social legitimacy are at stake.

In this sense, partial openness functions as a form of institutional mediation. It neither assumes that firms will internalise ethical demands voluntarily, nor presumes that all socially relevant interests can be protected through unrestricted access alone. Instead, it seeks to create governance arrangements under which ethically relevant forms of openness become structured, reviewable, and durable. This is precisely the kind of order-oriented solution that institutional economic ethics points toward.

The following and final section reflects on the policy implications of this model and asks how such a differentiated governance regime might relate to emerging regulatory frameworks for artificial intelligence.

## Policy Implications and Conclusion

### Policy Implications

The analysis developed in this paper has several implications for the governance of large language models. Most importantly, it suggests that ethical concerns surrounding LLMs should not be addressed solely through broad normative principles or through expectations of voluntary corporate responsibility. If the central problems of linguistic inequality, opacity, and contested data use are structurally linked to competitive incentives, then governance must focus on institutional design. In other words, the relevant task is not merely to formulate ethical standards, but to create rules and oversight structures that make responsible conduct more stable under real-world market conditions (Homann & Suchanek, 2005; Cuéllar & Huq, 2022).

A first implication concerns the design of transparency obligations. The argument of this paper does not support a simplistic demand for complete openness in all respects. Instead, it points toward differentiated forms of disclosure. Public institutions and regulators should require structured transparency regarding model documentation, known limitations, evaluation procedures, and data governance practices, while recognising that not every component of a model needs to be released under the same conditions. This suggests a layered regulatory approach in which different actors are granted different forms of access depending on their function. Public reporting, independent auditing, regulator access, and licensed research access need not be identical, but they should be institutionally specified rather than left to ad hoc corporate discretion (Berengueres, 2024; Chun & Elkins, 2024).

A second implication concerns the governance of data and provenance. If data acquisition and model performance are closely tied to competitive pressure, then legal and ethical uncertainty surrounding training data will not disappear through voluntary self-regulation alone. Governance frameworks should therefore require stronger documentation of data sources, categories, filtering procedures, and major limitations. The goal is not necessarily full public disclosure of all data, which may be infeasible or inappropriate in many cases, but rather institutional arrangements that make data governance more reviewable and contestable. This is especially important because questions of data provenance affect not only legality, but also fairness, legitimacy, and the social acceptability of large-scale AI development (Daly et al., 2021; Zhang et al., 2023; Hastings Blow et al., 2024).

A third implication concerns democratic oversight. The development and deployment of large language models increasingly affect infrastructures of communication, administration, and public knowledge. For that reason, governance cannot remain confined to bilateral relations between private developers and consumers. It must include institutional forms of public accountability. This may involve regulatory access to system information, requirements for third-party audits, reporting duties in high-impact domains, and stronger mechanisms for contestation and public review. Recent scholarship on democratic regulation and public governance in AI provides important support for this direction by stressing that legitimacy in AI governance depends on more than technical performance or corporate promises; it depends on the availability of institutional pathways for oversight and control (Bogiatzis-Gibbons, 2024; Manias et al., 2023; Cuéllar & Huq, 2022).

A fourth implication concerns competition and industrial organisation. The paper has argued that many ethical deficits associated with LLMs are reinforced by the concentration of resources and strategic advantage among a relatively small number of firms. This means that governance should not treat ethical and market questions as separate domains. Concerns about concentration, barriers to entry, and control over AI infrastructures are also concerns about the conditions under which socially desirable alternatives can emerge. A partially open governance model may therefore help not only by improving transparency, but also by lowering some barriers to participation and scrutiny without requiring the unconditional abandonment of all proprietary rights. In this respect, governance must remain attentive to the interaction between industrial organisation, innovation incentives, and accountability (Varian, 2018; Agrawal et al., 2019).

Taken together, these implications support a regulatory orientation that is neither purely prohibitive nor naively permissive. A governance regime for large language models should not simply attempt to stop innovation, nor should it rely on the assumption that innovation will regulate itself. The more plausible path lies in institutionally differentiated arrangements that allow innovation to continue while strengthening transparency, reviewability, and public legitimacy. This is precisely the rationale behind the proposal of partially open AI governance advanced in this paper.

### Conclusion

This paper has examined the ethical challenges of large language models through the lens of institutional economic ethics. Its central argument has been that many of the most pressing problems associated with LLMs—especially linguistic inequality, opacity, and contested data practices—cannot be understood adequately if they are treated only as matters of individual responsibility or technical imperfection. They are also products of an institutional environment in which firms operate under strong competitive incentives to scale rapidly, protect strategic assets, and prioritise commercially rewarding forms of development.

Karl Homann’s institutional economic ethics provides a useful framework for analysing this situation because it highlights the limits of moral appeals under conditions of competition. Ethical outcomes in markets cannot be secured reliably by assuming that firms will act against their own strategic interests whenever doing so is socially desirable. Instead, governance must focus on the rules of the game. In the field of large language models, this means that ethical AI governance should be understood as a problem of institutional design: a problem of how incentives, rights, obligations, and forms of access are structured.

On this basis, the paper has argued that the conventional opposition between open and closed AI systems is insufficient. Full closure may protect innovation rents and support control over deployment, but it also deepens opacity and weakens public accountability. Full openness may broaden participation and scrutiny, but it may also generate risks and undermine some incentives for private investment. For these reasons, a more differentiated approach is needed. The concept of partially open AI governance developed here aims to provide such an approach by distinguishing among different layers of AI systems and by proposing that access and disclosure be structured according to their ethical and economic functions rather than through an all-or-nothing model.

The contribution of the paper is therefore threefold. First, it applies Homann’s framework to the governance of large language models and shows how institutional economic ethics can deepen current debates in AI ethics. Second, it demonstrates that key ethical challenges of LLMs are closely linked to the political economy of AI development rather than merely to isolated technical defects. Third, it develops the concept of partially open governance as a more realistic and normatively defensible response to the tension between innovation incentives and public accountability.

The broader implication is that responsible AI governance requires more than ethical principles and more than market dynamism. It requires institutions capable of mediating between them. If large language models are becoming foundational infrastructures of communication and knowledge, then their governance must be organised in ways that make innovation compatible with public responsibility. A partially open governance regime does not resolve every tension in this field, but it offers a more plausible starting point for doing so than the false choice between unrestricted openness and opaque proprietary control.

## Data Availability

Data sharing is not applicable to this article as no datasets were generated or analyzed during the current study.

## Declarations

### Use of AI Tools

This manuscript benefited from AI-assisted language editing (ChatGPT and Google Translate), limited to copy editing. All substantive content is the responsibility of the authors.
